# Physicians' Attitude towards The Use of Social Media for Professional Purposes in Saudi Arabia

**DOI:** 10.1155/2019/6323962

**Published:** 2019-12-02

**Authors:** Turki Alanzi, Susan Al-Yami

**Affiliations:** Health Information Management and Technology Department, College of Public Health, Imam Abdulrahman Bin Faisal University, Dammam, Saudi Arabia

## Abstract

**Purpose:**

In relation to this research, only a few studies have been carried out around the world. However, in Saudi Arabia, there have been no investigations into this subject. In this sense, the objective of this study was to investigate the physicians' attitudes towards the use of social media for professional purposes in Saudi Arabia.

**Methods:**

A cross-sectional study was conducted among 235 physicians from different regions of Saudi Arabia. The data were collected by means of a survey. The survey questionnaire was distributed using the WhatsApp application. Descriptive statistics were used to analyze the results.

**Results:**

The most common social media used by the respondents was Facebook, and the majority of the participants agreed that social media improved their knowledge and skills. However, most of the participants did not interact with patients using these tools, did not feel comfortable conducting an online consultation, and believed that social media affected the choice of the healthcare provider. Similarly, 30.6% of the respondents opined that it is not appropriate to search online information about patients, and 44.3% of them considered that patients would not trust the medical advice if a physician obtained the information from a website.

**Conclusion:**

The results showed that the majority of the respondents considered that social media improved the knowledge and abilities of physicians. In addition, the respondents reflected that there were ethical issues that must be taken into account when using social media and more than half of them did not interact with their patients employing these tools. The outcomes of this research will help to develop programs directed at the physicians in Saudi Arabia to enhance their knowledge, professional skills, medicine practice, patient-doctor interaction, and handle the risks involved in the use of social media.

## 1. Introduction

The use of social media in almost all human activities is growing every day and has reached unprecedented statistics worldwide. In this regard, for example, the number of users of Facebook, YouTube, WhatsApp, Facebook Messenger, Instagram, Twitter, Skype, and Snapchat by April 2018 were 2234, 1500, 1300, 813, 330, 300, and 255 million people, respectively [1].

This increasing use of social media as a platform for universal communication has also reached the field of healthcare and has become a tool that doctors and other health professionals use to participate, share opinions, photos, audios, videos, and collect beneficial information to improve their professional knowledge and provide care to their patients [2–6]. Also, the use of social messaging applications and telemedicine websites has allowed patients to increase knowledge and education about their diseases, share their experience, and receive medical treatment, without the need for face-to-face appointments with their doctors [7–11]. In general, these social networking sites offer a variety of features that serve for different purposes according to the needs of users and can include blogs, social networks, video and photo exchange sites, wikis, and other dissemination alternatives. In relation to these general topics, several authors have studied the utilization of social media by doctors, students, and patients, emphasizing their uses, benefits, risks, barriers, and ethical and legal aspects [4, 12–15].

On the other hand, considering the objectives of our research, some studies have analyzed the opinions and attitudes of doctors on the use of social media in different fields of medicine [2–5, 15–19]. In the same way, in previous studies, the use of social media platforms has been considered as efficient instruments for education, exchange of ideas, and communication of experiences among physicians [3, 20, 21]. Additionally, some authors have analyzed the benefits, risks, potential challenges, and ethical and legal dilemmas that imply the management of these tools for doctors, nurses, and other healthcare professionals [3–5, 15, 17, 18, 22].

In another context, it should be noted that in the literature there are models that allow evaluating the attitude of people towards the use of specific technologies. One of these models is the technology acceptance model (TAM), which according to some recent versions considers that the attitude of people towards the use of a certain technology depends on the ease of use, availability, benefits, risks, and other factors [23]. This model has been used in some studies related to the use of social media and could be adapted to the subject of our research [23–26].

As we have seen, in different countries of the world, a few investigations have been conducted on the attitudes and opinions of physicians about the use of social media in the healthcare field; however, in Saudi Arabia, to the knowledge of the author, no research has been carried out on this topic, despite the massive use of social media in this region, where the penetration percentages of WhatsApp use, YouTube, Facebook, Instagram, and Twitter by 2017 have reached figures of 73%, 71%, 66%, 54%, and 52%, respectively [27].

In this sense, the objective of this research was to describe the physicians' attitude towards the use of social media for professional purposes in Saudi Arabia. The outcomes of this research will permit to develop programs directed to enhance knowledge, professional skills, medical practice, and patient–doctor interaction among physicians in the Kingdom of Saudi Arabia.

## 2. Methods

### 2.1. Study Settings and Participants

In order to assess the attitude of physicians towards the use of social media for professional purposes in Saudi Arabia, which is the object of this research, a cross-sectional study was carried out among physicians who practice in different regions of this country. Subjects were selected through the convenient nonprobability sampling method, and data were collected by means of a survey designed for such a purpose [28]. The population selected in this research consisted of 300 physician users of social media who were members of different WhatsApp groups in Saudi Arabia. This population was easily accessible, had experience in social media practices, and could provide adequate information on the research questions. All this population was contacted and the survey questionnaire was sent to them. Of this population, 235 participants filled out and returned the questionnaire. In other words, the response rate was 78.33%.

### 2.2. Description of the Questionnaire

The survey questionnaire contained 16 questions and it was designed using the Google forms provided in this link [29]. The survey questionnaire for this research was created based on surveys published in the literature and the experience of the researchers involved in this work [30]. To validate the study instrument, the questionnaire was sent to a small group of doctors who were users of social media in order to see if the questions were well-formulated and allowed to obtain the answers to the research questions. They filled out and returned the questionnaire through WhatsApp.

From the questions posed in the first part of the questionnaire (5 questions), the demographic information of the participants was obtained (age, gender, region, experience, and work position). Then, from the second part of the questionnaire consisting of the following 5 questions it is possible to know the types of social media used by the respondents, and how they employed these media for the medical treatment of their patients: which social media do you use at least once a week?; how many hours do you use social media per day?; as a physician do you interact with patients via email or social media?; which type of online media does your workplace currently have for the patient to access?; and do you ever discuss internet or social media usage with your patients?. Finally, from the 6 questions of the third part of the questionnaire, we got the opinions of the participants about the use of social media in various topics of their professional career: do you think social media can help to improve your knowledge and skills in your career?; do physicians have a duty to rebut inappropriate or inaccurate health information posted online?; if a physician obtained the medical information from a related mobile phone application or website, would a patient trust medical advice?; is it appropriate for physicians to look up any available online information about a patient as part of regular clinical practice?; would you be comfortable conducting a consultation with a patient via skype or other online telecommunications?; and would social media affect the patients choice of the healthcare provider?

It is pertinent to clarify that in the above questions we can detect the independent and dependent variables of our research knowing that the dependent variables respond to the independent variable. In other words, the independent variables are associated with the cause and the dependent ones to the effect. In our case, the dependent variables are the responses of the participants expressed in percentages. As an example, in the question; Do you think that social media can help to improve your knowledge and skills in your area? The independent variable is “social media”, and the dependent variable is “knowledge and skills”, and so on with the rest of the questions.

### 2.3. Data Collection

The data required for evaluating the physicians' attitude towards the use of social media in Saudi Arabia were collected using a survey. The survey questionnaire was distributed to 300 physicians of different categories from interns to consultants who practice in this region using the WhatsApp application. From this sample, we received responses from 235 physicians.

### 2.4. Statistical Analysis

Descriptive statistics in terms of frequency and percentage were used to analyze the results of this study. Also, the significance between the demographic variables and the attitude of physicians was tested by means of the Chi-square test. The analysis was done using the SPSS 21.0 version and *p*-values less than 0.05 were considered as significant.

## 3. Results

The demographic data of the 235 respondents are shown in Table 1. According to this table, most of the participants (72.3%) were female, more than half of the respondents (54.4%) were less than 35 years old, and the majority of them (70.2%) belonged to the eastern region of Saudi Arabia. Also, 34.0% of the respondents were consultant and 33.6% were resident. Similarly, 56.1% of the surveyed physicians have less than 10 years of experience.

Table 2 presents the questions and responses of the participants about the use of social media. In a descending order, the respondents used the following platforms: Facebook (37.9%), YouTube (35.7%), Twitter (34.5%), Google+ (17.9%), Research gate (9.8%), LinkedIn (7.7%), and Instagram (5.9%). Also, 46.4% of the workplaces utilized text message for patients' access, and almost half of the participants (45.5%) spent 2 to 3 hours per day working on social media platforms. Most of the surveyed physicians (67.3%) did not interact with patients via e-mail or social media. In addition, more than half of the participants (57.5%) did not discuss internet or social media usage with patients.

The questions and the participants' opinions about the use of social media are described in Table 3. This table shows that 76% of respondents agreed that social media enable them to improve their knowledge and skills, and 70% of them thought that physicians have a duty to rebut inappropriate or inaccurate health information posted online. Similarly, 30.6% of respondents disagreed that it is appropriate for physicians to look up any available online information about a patient as part of regular clinical practice, and 44.3% of participants disagreed that a patient would trust the medical advice if a physician obtained the medical information from a related social media website. On the other hand, more than half of surveyed, 53%, did not feel comfortable conducting a consultation with a patient using online telecommunications. In addition, most of the respondents (74.0%) stated that social media will affect the patients choice of the healthcare provider.

Table 4, applying the Chi-square test shows the statistical importance of the respondent's age and the type of social networks used. Similarly, the statistical significance between the demographic variables of the respondents and the role of social media to improve knowledge and skills is presented in Table 5 using the probability value found by the Chi-square test.

## 4. Discussion

The findings of this study about the physicians' attitudes towards the use of different types of social media in Saudi Arabia indicated that the most used social media platforms by the participants were in decreasing order: Facebook (37.9%), YouTube (35.7%), Twitter (34.5%), Google+ (17.9%), Research gate (9.8%), LinkedIn (7.7%), and Instagram (3.9%). In addition, 90.5% of participants utilized these social media tools between 1 and 3 hours a day. In this regard, in a survey carried out with participants from 29 countries it was found that more than half of the participants considered that Facebook was more useful than Twitter for professional purposes; this finding coincides with the general tendency observed in our research [31]. Similarly, Facebook was reported as the most used social media platform (59.9%) by Australian doctors [30].

In our study, we detected that 76% of the participants believed that social media contributes to improving the knowledge and skills of the participants. About this topic, previous studies have suggested the utility of social media as an educational tool for medical professionals [3, 20, 21].

Our results also showed that more than half of the participants (67.3%) did not interact with patients using social media, and 57.5% of the participants did not discuss with patients the social media or the Internet usage. In relation to this matter, similar results have been observed in other investigation in which more than half of the respondents (59%) were opposed to employing social media for patient interaction [32].

About the information published online, we observed that 70% of the respondents considered that the doctors have the duty to refute any inappropriate information published on social networks. Alike, 30.6% of respondents thought that it is not appropriate for physicians to look up any patient information available online; however, 33.2% of respondents had an opposite opinion to this concern; and 36.2% had a neutral attitude on this matter; regarding this topic, several authors have considered the importance of maintaining the confidentiality of patients [12–15, 33, 34].

Additionally, we appreciated that 44.3% of the participants believed that a patient would not trust the advice of a doctor if he takes medical information from social media; regarding this issue, 18.7% presented an opposing opinion to this concern, and 37% had an impartial judgment. As well, more than half of the respondents (53%) did not feel comfortable conducting a consultation with a patient online; a similar response is observed in another research in which 60.8% of respondents did not feel comfortable interacting online with their patients [30]. Besides, the majority of respondents (74%) opined that social media affects the selection of the healthcare provider.

On the other hand, it is possible to infer that there was an association between age and the type of social media used since the p-values calculated using the Chi-square test were less than 0.05. According to this information, we appreciated that most of the young people (25–35 years old) used Facebook, preferably; and, the older ones (56–65 years old) mostly utilized Research gate.

Correspondingly, the calculated p-value employing the Chi-square test suggested that there was a statistically significant relationship between the age of the respondents and the role of social media to improve knowledge and skills in the professional career. In this sense, in contrast to most of the young participants, the older respondents felt that social media information did not contribute significantly to improve knowledge and professional skills. Additionally, no significant statistical relationship was observed between the gender, experience, position, and the impact of social media to improve knowledge and professional skills.

It is pertinent to point out that the main limitation of this work was the small sample size of participants in comparison with the number of doctors in Saudi Arabia which limits its general validity. Also, since the convenient non probability sampling method was used in this study, the results found are only valid for the sample of the 235 respondents of social media users belonging to the different WhatsApp groups in Saudi Arabia who answered and filled out the questionnaire of the survey. The results cannot be generalized to the total population of doctors in Saudi Arabia. In this regard, to obtain information that allows generalizing the results to the entire population, it is necessary to increase the sample size since the total population of doctors living in Saudi Arabia was about 10,000 in 2018 [35]. Therefore, for future studies, a larger sample size is recommended with a cluster sampling that includes the different geographical regions of Saudi Arabia. Also, it is convenient to conduct studies to examine the differences of opinion among physicians and other categories of health professionals about the use of social networks to share and exchange medical knowledge. Likewise, it would be convenient to conduct an investigation into the differences in the uses of social media among doctors in Saudi Arabia and other places in the world. Similarly, it would be interesting to investigate whether there are cultural, technical, or practical reasons that limit the use of social media for the professional training and education of doctors in this country. Additionally, it would be useful to carry out a qualitative analysis about the thinking of physicians and to contextualize this study within a theoretical framework such as the TAM model that could help to explain the attitudes of physicians towards the use of social media for professional purposes in Saudi Arabia.

## 5. Conclusion

The results of this research showed that the most used social by the surveyed physicians was Facebook and the majority of respondents believed that social media offer and disseminate scientific and technological information through articles, books, forums, case studies, videos, and other systems that contribute to improving the knowledge and development of the professional skills of doctors.

However, most of the respondents did not interact with patients and thought that there were ethical dilemmas related to the privacy of patients and the publication of inappropriate information that should be taken into account in the use of social media. Finally, the outcomes of this investigation will allow creating programs focused to improve knowledge, professional skills, medicine practice, patient-doctor interaction, and handle the risks involved in the use of social media among physicians in the Kingdom of Saudi Arabia.

## Figures and Tables

**Table 1:** Demographic data of the respondents (*n* = 235).

Variables	*n*	%
*Age in years*		
Less than 25	25	10.6
25–35	103	43.8
36–45	38	16.2
46–55	46	19.6
56–65	23	9.8

*Gender*
Male	64	27.2
Female	171	72.3

*Region*
Center	45	19.1
East	165	70.2
North	2	0.8
South	12	5.1
West	11	4.8

*Experience in years*
0–2	77	32.7
3–5	24	10.2
6–10	31	13.2
More than 10	102	43.4

*Position*
Consultant	80	34.0
Resident	79	33.6
Specialist	38	16.2
Interns	29	12.3
Others	9	3.8

**Table 2:** Questions and responses about the type and ways of using social media (*n* = 235).

Questions	*n*	%
*Which social media do you use at least once a week?*		
Facebook	89	37.9
YouTube	84	35.7
Twitter	81	34.5
Google+	42	17.9
Research gate	23	9.8
LinkedIn	18	7.7
Instagram	14	5.9

*How many hours do you use social media per day?*		
Never	3	1.3
0–1	69	29.4
2–3	107	45.5
4–6	36	15.6
More than 6	20	8.5

*As a physician do you interact with patients via email or social media?*		
Yes	77	32.7
No	158	67.3

*Which type of online media does your work place currently have for patient to access?*		
Website	52	22.1
Text message	109	46.4
Instagram	5	2.1
Facebook	12	5.1
Others	49	24.3

*Do you ever discuss internet or social media usage with your patients?*		
Yes	100	42.5
No	135	57.5

**Table 3:** Questions and opinions about the use of social media (*n* = 235).

Questions	%
*Do you think social media can help to improve your knowledge and skills in your career?*	
Yes	76
No	12
Unsure	12

*Do physicians have a duty to rebutt inappropriate or inaccurate health information posted online?*	
Yes	70
No	12
Unsure	18

*Is it appropriate for physicians to look up any available online information about a patient as part of regular clinical practice?*	
Agree	33.2
Neutral	36.2
Disagree	30.6

*If a physician obtained the medical information from a related mobile phone application or website, would a patient trust medical advice?*	
Agree	18.7
Neutral	37
Disagree	44.3

*Would you be comfortable conducting a consultation with a patient via skype or other online telecommunications?*	
Yes	24
No	53
Unsure	23

*Would social media affect the patients choice of healthcare provider ?*	
Agree	74
Neutral	22.5
Disagree	3.4

**Table 4:** Statistical significance between the respondents' age and the type of social media usage (Chi-square test).

Type of social media	Age group in years					*P* value
	Less than 25	25–35	36–45	46–55	56–65	
Facebook	23	56	6	3	1	0.021
Twitter	13	20	34	12	2	
Google+	15	20	4	2	1	
LinkedIn	3	6	2	6	1	
YouTube	10	17	19	21	17	
Instagram	6	2	0	2	4	
Research gate	2	3	6	2	10	

**Table 5:** Statistical significance between the demographic variables of the respondents and the role of social media to improve knowledge and skills in career (Chi-square test).

Variables	Yes (%)	*P* value
*Age*		
Less than 25	25	<0.001
25–35	103	
36–45	38	
46–55	46	
56–65	23	

*Gender*
Male	64	0.567
Female	171	

*Experience in years*
0–2	77	0.432
3–5	22	
6–10	34	
More than 10	102	

*Position*
Consultant	80	0.703
Resident	61	
Specialist	39	
GP	19	
Interns	29	
Others	7	

## Data Availability

The data used to support the findings of this study are available from the corresponding upon request.
